# Post–EBV Cold Urticaria with High-Titre Cold Agglutinins without Haemolysis: A Paediatric Case Report

**DOI:** 10.31138/mjr.221025.rcu

**Published:** 2026-06-01

**Authors:** Zoe Bezirgiannidou, Iliana Stamatiou, Christina Spanopoulou, Antigoni Mavroudi, Nikolaos Kitsos, Emmanouil Spanoudakis, Ioannis Kotsianidis, Dimitrios Cassimos

**Affiliations:** 1 Department of Haematology, University Hospital of Alexandroupolis, Democritus University of Thrace, Alexandroupolis, Greece;; 2 Blood Transfusion, University Hospital of Alexandroupolis, Alexandroupolis, Greece;; 3 Department of Rheumatology, KAT General Hospital of Athens, Athens, Greece;; 4 Faculty of Medicine, Aristotle University of Thessaloniki, Thessaloniki, Greece;; 5 Department of Paediatrics, Faculty of Medicine, University of Thessaly, Larissa, Greece;; 6 Paediatric Department, University Hospital of Alexandroupolis, Democritus University of Thrace, Alexandroupolis, Greece

**Keywords:** cold urticaria, cold agglutinins, Epstein–Barr virus, paediatrics, immune response

## Abstract

This report describes a paediatric case of cold urticaria (ColdU) associated with markedly elevated cold agglutinin titres following Epstein–Barr virus (EBV) infection. An 11-year-old girl developed recurrent cold-induced urticaria and anaphylaxis after a recent EBV infection. Immunohaematologic testing revealed a cold-reactive IgM autoantibody with anti-i specificity. Despite the exceptionally high peak titres (>1:262,144 at 4°C), and the unusually broad thermal amplitude extending to 30°C, no clinical or laboratory evidence of haemolysis was observed, and the direct antiglobulin test remained persistently negative. Complement components and cryoprotein assays were consistently normal. Throughout an extended 52-month longitudinal follow-up, antibody titres gradually declined in parallel with the resolution of urticarial symptoms and withdrawal of antihistamines. This case demonstrates a non-haemolytic, post-infectious immune phenotype of ColdU, in which transient, high-titre cold agglutinins coexist with cutaneous hypersensitivity without haemolytic manifestations, and highlights the importance of comprehensive immunohaematologic evaluation and long-term monitoring in ColdU cases with atypical serologic findings.

## BACKGROUND

Cold urticaria (ColdU), previously referred to as cold-induced urticaria (CIU), was first reported in 1866^1^ and is now classified among chronic inducible urticarias according to guidelines.^2^ Patients present with pruritic wheals and angioedema after exposure to cold stimuli, including cold air, water, or objects. Anaphylaxis is observed in one-third of cases, with or without respiratory distress and hypotension.^3^ These immediate or delayed reactions result from histamine and leukotrienes release triggered by cold exposure, with mast-cell degranulation being the key event, although the exact pathogenesis of ColdU remains unclear.^3^ Autoantibodies, cold-inducible immunoglobulin E (IgE), or occasionally immunoglobulin M (IgM) may trigger mast cell activation although no specific antigen has been defined. The recommended laboratory workup includes a complete and differential blood count, erythrocyte sedimentation rate, total serum IgE, complement levels, infectious serologies, liver function tests, and cryoprotein studies—encompassing cryoglobulins, cryofibrinogen, cold agglutinins, and biphasic haemolysins such as Donath–Landsteiner antibodies.^5^ Cold agglutinins and cryoglobulins have been found in a substantial subset of ColdU patients and may correlate with disease severity or chronicity. In a prospective cohort involving 35 patients with cold urticaria, 46% and 27% tested positive for cold agglutinins and cryoglobulins, respectively, with cold agglutinin positivity linked to cold-air or cold-water triggers and lower disease control.^4^ Cryoglobulin levels correlated with both serum tryptase concentrations and disease duration, suggesting a potential pathogenetic role.^4^

In the majority of patients, the disease tends to follow a chronic course, with symptoms lasting for several years, regardless of onset age at onset.^5,6^ While most cases of ColdU are idiopathic, some may arise due to an underlying condition, primarily cryoglobulinemia or cold agglutinin disease (CAD).^6,7^ Notably, immune reactivity involving cold agglutinin can occur without clinical haemolysis, suggesting that immunologic mechanisms may be active beyond red blood cell pathology.^8^ In this context, we report a paediatric case of ColdU, following EBV infection, presenting with unusually high-titre cold agglutinins with broad thermal amplitude, no haemolytic activity and persistently negative direct antiglobulin test (DAT) for anti-C3d.

## CASE PRESENTATION

An 11-year-old female patient was evaluated for recurrent episodes of urticaria at the Allergy Clinic of our hospital. Symptoms were consistently provoked by cold air or water exposure. One of the episodes was complicated by anaphylaxis; however, no relapses followed food or inhalant allergens exposure. Physical examination was normal. The ice cube test was positive and reproducible after skin rewarming.

Laboratory evaluation showed a haemoglobin level of 14.1 g/dL, white-cell count of 7.07×109/L, platelet count of 365×109/L, mean corpuscular volume (MCV) 78.5 fL (79–98), lactate dehydrogenase (LDH) level of 177 IU/L (reference range: 134–279), creatinine 0.6 mg/dL (0.6–1.2), alanine aminotransferase (ALT) 84 IU/L (0–55), aspartate aminotransferase (AST) 20 (5–34), and total bilirubin 0.6 mg/dL (0.2–1.2) with direct bilirubin 0.1 mg/dL (<0.5). The reticulocyte count was 0.97% (0.5–2.0). The coagulation studies revealed a normal international normalised ratio of 0.9 (0.8–1.2) and a normal fibrinogen level (309 mg/dL). The serum haptoglobin levels were also within normal limits (<5 mg/dL).

Serologic testing for infectious diseases revealed markedly elevated anti–Epstein–Barr virus (EBV) IgG antibody titres (>1000 AU/mL), while EBV IgM antibodies were within the normal range, a pattern compatible with a recent primary EBV infection captured during the early convalescent phase. At follow-up three months later, EBV IgM antibodies remained negative, while EBV-specific IgG antibodies persisted with declining titres. Subsequent tests demonstrated IgG positivity to parainfluenza types 1–3, whereas IgM tests remain negative, as well as mildly elevated antistreptolysin-O titres. Routine immunological markers such as ANA, complement components C3 and C4, and immunoglobulin levels of IgA, IgG, and IgM, remained within the normal range. The IgE level of 4.01 IU/mL falls within the range of 0.1–100, suggesting no substantial allergic or parasitic trigger. Based on the typical clinical history and the positive ice cube test, the diagnosis of ColdU was confirmed according to EAACI/GA^2^LEN/EDF/WAO guidelines,^2^ and an extended immunohaematologic evaluation was scheduled to evaluate the presence of a cold-reactive autoantibody.

## IMMUNOHAEMATOLOGIC INVESTIGATION

Direct antiglobulin testing (DAT) remained negative throughout the disease course, including monospecific testing for IgG, IgA, IgM, and complement (C3c/C3d); DAT also remained negative under cold-stress conditions at 4°C,^9^ indicating absence of in vivo complement activation. Indirect antiglobulin testing (IAT) was likewise negative at all evaluated temperatures (4°C, 22°C, and 37°C). Acid and cold acid elution studies were negative, supporting the absence of clinically relevant RBC-bound antibody reactivity. Cryoprotein-related testing was negative (including cryoglobulins and cryofibrinogen), and evaluation for paroxysmal cold haemoglobinuria (PCH) with a Donath–Landsteiner test was also negative. Importantly, these investigations—including DAT and IAT—were repeated at follow-up assessments (0, 3, 4, 6, 7, 17, and 52 months) and remained consistently negative, with no clinical or laboratory evidence of haemolysis.

## COLD AGGLUTININ TITRATION

**Figure 1.**
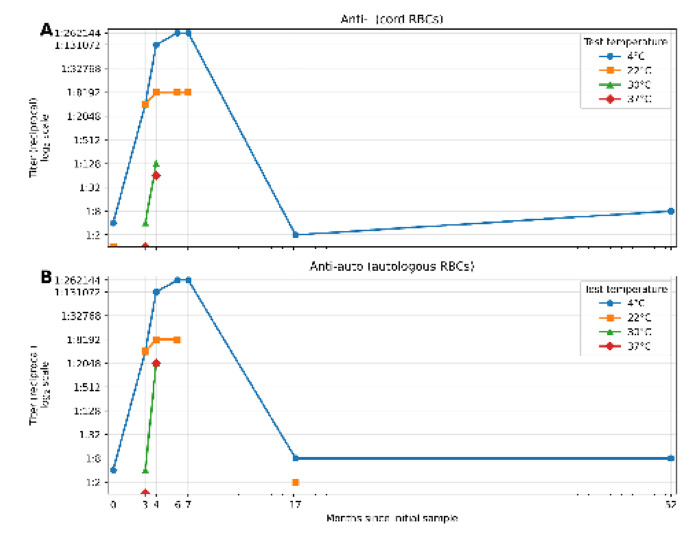
**Cold agglutinin titres over time and thermal amplitude.** Cold agglutinin titrations were performed by the classical tube method at 4°C, 22°C, 30°C, and 37°C. The x-axis shows months since the initial sample (0, 3, 4, 6, 7, 17, and 52), and the y-axis displays reciprocal titres on a log₂ scale (two-fold serial dilutions). Timepoints tested with no macroscopic agglutination (titre = 0) are plotted at 1:1 (reciprocal titre = 1) and shown as isolated markers (not connected by lines). Panel A (A): anti-i reactivity tested against cord RBCs (i^+^). Panel B (B): autoagglutination tested against autologous RBCs. **Note:** Auto-RBC agglutination detected at 30–37°C is compatible with a high-titre, broad–thermal amplitude IgM cold agglutinin and does not imply a warm (IgG) autoantibody.

Additional investigation therefore focused on serial cold agglutinin titration, performed by the classical tube method at 4°C, 22°C, 30°C, and 37°C, with macroscopic assessment of agglutination. Thermal amplitude and apparent antigen specificity were evaluated across an expanded RBC target panel, including cord O(i) RBCs, autologous A1 RBCs, adult O(I) RBCs (anti-H target), adult A1 and A2 RBCs, and enzyme-treated adult O(I) RBCs. Overall kinetics were similar across targets, but the highest titres were most consistently observed against cord (i^+^) RBCs and autologous A1 RBCs. **Figure 1** depicts the longitudinal titre evolution against these two targets for clarity.

At the first evaluation following symptom onset (month 0), cold agglutinin reactivity was minimal and limited to weak agglutination at 4°C (1:4), with no reactivity at higher temperatures. By month 3, titres rose to 1:4096 at 4°C and 1:2048 at 22°C (against both cord and autologous RBCs), and increased further during the symptomatic phase: at month 4, anti-i titres reached 1:131,072 at 4°C, and at months 6–7 titres at 4°C exceeded 1:262,144. By later follow-up (months 17 and 52), titres had declined to low-level reactivity confined to 4°C (approximately 1:2–1:8). The longitudinal evolution of titres across temperatures and RBC targets is shown in **Figure 1**.

Peak-phase serum treated with 1,4-dithiothreitol (DTT), a sulfhydryl reducing agent that disrupts IgM pentamers by reducing disulfide bonds,^11^ produced a marked reduction in titre (e.g., >1:262,144 to 1:512 at 4°C), supporting IgM-mediated cold agglutinin activity. At peak activity, agglutination was detectable also at 37°C, but the corresponding titre was much lower than the concomitant cold-phase titre (e.g., ~1:2048 at 37°C vs 1:131,072 at 4°C). This pattern is consistent with broad thermal amplitude in vitro and, together with persistently negative DAT/IAT and the absence of in vivo haemolysis, does not necessarily indicate clinically pathogenic warm autoimmunity. Additional technical details and complete tabulated titration results are provided in the Supplementary material. The laboratory improvement paralleled patient’s clinical stability, supporting the interpretation that the autoantibody response was both transient and self-resolving. Despite high cold agglutinin titres and broad thermal activity, no signs of in vivo haemolysis occurred, as summarised in **Table 1.**

**Table 1.** Longitudinal immunohaematologic and haematologic laboratory findings.

**Follow-up month**	**Cold agglutinin titre**	**Thermal amplitude**	**IAT**	**DAT**	**Hb (g/dL)**	**WBC (×10^9^/L )**	**PLT (×10^9^/L )**	**LDH (IU/L)**	**Total bilirubin (mg/dL)**	**Haptoglobin (mg/dL)**
0	1:4	4°C only	Negative	Negative	14.1	7.07	365	177	0.6	<5
~3	1:4,096	up to 22°C	Negative	Negative	13.4	4.62	332	165	0.5	<5
~4	1:131,072	up to 30– 37°C	Negative	Negative	13.2	5.44	349	171	0.4	<5
~7	>1:262,144	up to 30– 37°C	Negative	Negative	14.7	6.10	309	168	0.6	<5
~17	1:128	4°C only	Negative	Negative	13.4	4.77	300	160	0.5	<5
~52	1:4	4°C only	Negative	Negative	13.6	5.20	309	162	0.4	<5

**Table 1** summarises the longitudinal cold agglutinin titre (reciprocal endpoint by classical tube testing, reported for cord O(i) RBCs/anti-i), the corresponding thermal amplitude (highest temperature with visible agglutination), and concurrent DAT/IAT and haemolysis-related laboratory parameters during follow-up.

## DISCUSSION

This case presents a unique case of paediatric ColdU linked to extremely high-titre cold agglutinins—without any evidence of haemolysis—following EBV infection.^8,12^ It underscores the need for comprehensive cold agglutinin determination, particularly in seronegative cases, and emphasises that cold autoantibodies, despite high titres and varied thermal amplitudes may reflect a post-infectious immune response.^8,13^

ColdU has been associated with several clinical conditions, including infectious mononucleosis, systemic vasculitis, connective tissue disorders, cryoglobulinemia, cryofibrinogenaemia, CAD, Waldenström’s macroglobulinemia, B-cell lymphoproliferative disorders, and paroxysmal nocturnal haemoglobinuria; however, a definitive causal relationship has not been established.^4^ The proposed pathophysiological mechanism typically involves complement activation triggered by circulating abnormal proteins, yet the precise link between these conditions and ColdU remains unclear.^4^

In our case, the co-occurrence of a cold-reactive autoantibody with anti-i specificity and the clinical presentation of ColdU may reflect an immunological consequence of prior EBV infection, which is known to modulate humoral immunity and induce transient autoantibody responses such as anti-i cold agglutinins.^8,12^ Although cytokine levels were not measured in the present case, previous studies have shown that elevated IL-6, TNF-α, and IFN-γ may enhance mast-cell reactivity and lower their activation threshold, promoting cold-induced urticarial responses.^4^ This combined immune response—marked by autoreactive B-cells and cytokine-sensitised mast cells—may clarify the co-stimulation of cold autoantibodies and urticaria, consistent with findings in paediatric EBV infections, particularly among patients retaining the foetal “i” antigen.^8,12^

Serological analysis, in this case, revealed a complex immune response. At initial screening, anti-EBV IgG titres were markedly elevated (>1000 AU/mL) while IgM were already undetectable, indicating a recent acute infection captured during the early convalescent phase. At the 3-month follow-up, EBV serology demonstrated persistent IgG positivity with declining titres (EBV VCA IgG: 850 AU/mL; EBNA IgG: 5.8 AU/mL), while IgM antibodies remained negative, a pattern compatible with ongoing convalescence after acute infection. This seronegative interval overlapped with the onset of cold agglutinin production and cold urticaria symptoms. Over time, both antibody titres and clinical manifestations declined in parallel, supporting a post-infectious immune phenomenon occurring shortly after acute EBV infection. This pattern aligns with previous observations linking transient cold agglutinin production during convalescence from EBV-related illness.^14^

A cold-reactive IgM antibody with anti-i specificity, was identified in this patient, exhibiting markedly elevated titres—exceeding 1:262,144 at 4°C—and measurable reactivity at 30°C and occasionally at 37°C, indicating a rare, broad thermal activity.^8,15^ Importantly, thermal amplitude is generally a more clinically informative determinant of haemolytic risk than titre alone, as it reflects the temperature range within which antibody– RBC binding may occur in vivo. These antibodies can trigger the classical complement pathway through C1q binding, leading C3 convertase formation, subsequent C3 cleavage, and C3b deposition on RBCs.^14^ This process typically facilitates opsonisation and extravascular haemolysis; whereas intravascular haemolysis may occur in cases of unregulated terminal pathway activation.^16^ In this patient, the DAT, including anti-C3d assessment, was persistently negative, and complement levels remained within normal ranges, suggesting that any level of activation of the classical pathway was inadequate to trigger detectable C3b deposition or clinically significant haemolysis.^13,16,17^

This discrepancy highlights the importance of factors beyond antibody titre and thermal amplitude in determine pathogenicity. One potential explanation may involve a limited complement-fixing capacity of the IgM antibodies, possibly related to reduced binding affinity, in combination with effective inhibition by erythrocyte-associated membrane-bound complement regulators such as CD55 and CD59.^15^ This aligns with recent findings showing that while classical complement pathway activation by cold agglutinins is relatively common, intact complement regulatory mechanisms often prevent progression to clinically significant haemolysis. Additionally, partial deficiencies in classical pathway components or impaired formation of the terminal complement complex (C5b–9) may contribute to the lack of haemolytic manifestations, though these mechanisms remain speculative. Supporting evidence comes from case reports describing high-titre cold-reactive IgM antibodies without haemolytic activity.^8,15,16^ In addition, other immune-related effects may contribute to the development of ColdU. Autoantibodies of IgM and IgG isotypes targeting either IgE or its high-affinity receptor, FcεRI, have been detected in affected patients.^18,19^ These autoantibodies can set off mast cell degranulation by cross-linking their receptors, effectively simulating exposure to allergens.^18^

Experiments involving the passive transfer of sera have confirmed the pathogenic nature of such sera.^18^ Although total serum IgE levels may appear normal, yet local IgE receptor occupancy can still induce mast cell hypersensitivity, supporting the role of tissue-level immune dysregulation in ColdU pathophysiology.^16,19^

Cold urticaria has also been associated with various infectious organisms, including *Toxoplasma gondii,* Epstein-Barr virus, hepatitis C, HIV*, and Helicobacter pylori.*^12,19–22^ Despite these associations, cryoproteins are rarely detected. Alangari et al. reported that cryoglobulins were absent in paediatric ColdU cases and present in less than 10% of adult cases.^7^ Consistent with our observations, Ginter et al. in a systematic review of 1,151 patients, identified cryoglobulins in just 3.0%, cold agglutinins in 1.1%, and cryofibrinogens in only 0.7%. In their prospective cohort of 49 patients, cold agglutinins were detected in only two patients (4.3%), neither of whom had evidence of an underlying autoimmune or hematologic disorder.^23^ In most otherwise healthy individuals, ColdU is therefore idiopathic, with post-infectious immune system activation being a more plausible cause than persistent cryoproteinaemia.^16^

The gradual improvement of urticaria symptoms and decline in cold agglutinin titre, along with the cessation of antihistamine treatment, supports a self-limiting immune response.^6^ This temporal relationship indicates that cold agglutinin production was a transient side effect of immune activation following EBV infection, rather than a manifestation of a clonal B-cell proliferation.^13^ Further investigation is needed to determine whether these antibodies directly trigger ColdU or represent a temporary immune response overreaction following a viral infection.^13,17^

This case highlights an uncommon immunological phenotype of ColdU characterised by high-titre cold agglutinins that remained clinically silent. These findings underscore that cold agglutinin serology and cold urticaria represent distinct pathogenic processes, with the former primarily involving complement-mediated erythrocyte injury and the latter reflecting a mast cell–driven cutaneous hypersensitivity response. Unlike most reports that emphasise haemolytic manifestations, our longitudinal findings provide longitudinal immunohaematologic evidence that cold autoantibodies can exist transiently without pathogenic consequences. This challenges the assumption that antibody titre and thermal amplitude alone define clinical relevance and highlights the importance of complement function assessment in diagnostic evaluation. To our knowledge, this report presents one of first paediatric cases with comprehensive antibody profiling and extended 52-month follow-up, providing new insights into post-infectious immune mechanisms in ColdU and the behaviour of non-haemolytic cold autoantibodies.

## CONCLUSION

To conclude, this case underscores the value of comprehensive immunohaematologic approach as well as a long-term follow-up in cold urticaria with atypical serologic features. The pathogenic significance of cold autoantibodies must be interpreted within a broader clinical and laboratory context, rather than inferred solely from titre or thermal reactivity.

## Data Availability

Data supporting the findings of this study are available from the corresponding author upon reasonable request.

## REPORTING GUIDELINES

This case report was prepared in accordance with the CARE (CAse REport) guidelines. CABARET principles were considered where applicable, without altering the case report design of the study.

## SUPPLEMENTARY APPENDIX

https://mjrheum.org/assets/files/supplementary/MJR-2025-0371_Supplementary-Appendix.pdf
